# 

**Published:** 1899-01

**Authors:** 


### CONTENTS.

ORIGINAL ARTICLES: PAGE

On the Relations which exist between Human Tuberculosis and Bovine Tuberculosis. By PROF. ED. NOCARD. Translated by MAZYCK P. RAVENAL, M.D. . . . 1

The Organism of Lung-plague. By LEONARD PEARSON, B.S., V.M.D. . . . 8

Glanders and its Suppression; Experiments with Mallein. By J. M. WRIGHT, M.D.C. . . . 12

A New Method of Employing Charcoal in the Treatment of Acute Indigestion in Horses. By GEORGE J. GOUBEAUD, D.V.S. . . . 16

Cribbing, and its Operative Treatment. By S. J. J. HARGER, V.M.D. . . . 22

Hypodermic Cathartics. By E. STANTON MUIR, Ph.G., V.M.D. . . . 26

DEPARTMENT OF SURGERY . . . 36

REPORTS OF CASES . . . 38

CORRESPONDENCE . . . 42

EDITORIALS . . . 45

NECROLOGY . . . 47

COLLEGE AND SOCIETY NOTES . . . 48

SOCIETY PROCEEDINGS . . . 50

PERSONALS . . . 62

